# Diversities and interactions of phages and bacteria in deep-sea sediments as revealed by metagenomics

**DOI:** 10.3389/fmicb.2023.1337146

**Published:** 2024-01-08

**Authors:** Xumei Sun, Haibo Jiang, Siyuan Zhang

**Affiliations:** School of Marine Sciences, Ningbo University, Ningbo, China

**Keywords:** deep-sea, sediments, phages, bacteria, interactions

## Abstract

Phages are found virtually everywhere, even in extreme environments, and are extremely diverse both in their virion structures and in their genomic content. They are thought to shape the taxonomic and functional composition of microbial communities as well as their stability. A number of studies on laboratory culture and viral metagenomic research provide deeper insights into the abundance, diversity, distribution, and interaction with hosts of phages across a wide range of ecosystems. Although most of these studies focus on easily accessible samples, such as soils, lakes, and shallow oceans, little is known about bathypelagic phages. In this study, through analyzing the 16S rRNA sequencing and viral metagenomic sequencing data of 25 samples collected from five different bathypelagic ecosystems, we detected a high diversity of bacteria and phages, particularly in the cold seep and hydrothermal vent ecosystems, which have stable chemical energy. The relative abundance of phages in these ecosystems was higher than in other three abyssal ecosystems. The low phage/host ratios obtained from host prediction were different from shallow ecosystems and indicated the prevalence of prophages, suggesting the complexity of phage–bacteria interactions in abyssal ecosystems. In the correlation analysis, we revealed several phages–bacteria interaction networks of potential ecological relevance. Our study contributes to a better understanding of the interactions between bathypelagic bacteria and their phages.

## Introduction

Viruses that infect bacteria (phages) are the most abundant and genetically diverse biological entities on Earth, infecting specific hosts in every known environment (Harada et al., 2018). Phages are ubiquitous and exhibit a plethora of morphologies, genetics, and phylogenies (Dion et al., 2020). Much of our knowledge of phage diversity has been overhauled following the advances in large-scale viral metagenomics and culturing efforts. Thousands of viral sequences have been identified from metagenomic sequencing projects; however, many of them share no detectable homology with reference phage genomes (Paez-Espino et al., 2016; Gregory et al., 2019). Examples of such sequences include the numerous discoveries of non-tail ssDNA or dsDNA phages (Kauffman et al., 2018). In addition to phage diversity, in recent years, growing numbers of studies have focused on the interactions of phages and bacteria in complex communities, such as oceans, soils, guts, among others (Goordial et al., 2017; Kutáková et al., 2018; Cornuault et al., 2020; Zhang et al., 2023). In the ocean, the crucial role of marine phages can be attributed to their tremendous abundance and diversity (Suttle, 2007). Phages, whether virulent or lysogenic, are considered a major force in shaping the composition of microbial communities, steering bacterial evolution, and holding key implications for biogeochemical cycles through bacterial mortality (Obeng et al., 2016; Chevallereau et al., 2022). Such interaction is universal in nature (van Houte et al., 2016; Calero-Cáceres et al., 2019). Diverse environments and biological compositions shape multiple phage–host interactions; for example, the presence of multiple phages influenced the mode and the structure of some environments reduced the coevolutionary rate due to the decreased contact between bacteria and phages (Betts et al., 2018; Lourenço et al., 2020). At present, most studies of phage–host interaction are based on laboratory culture or sequencing of samples that are easily obtained. However, there is limited research on phage–host interactions in areas that are challenging to access, such as the deep sea.

The deep-sea ecosystem, one of the most important but extreme ecosystems on the Earth, occupies approximately two-thirds of the Earth's surface (Orcutt et al., 2011). Generally, the deep sea is considered to be the area below the mesopelagic zone, which possesses the largest aqueous habitat for life (Kobayashi et al., 2012; Reygondeau et al., 2018). According to different environmental characteristics and formation, the deep sea can be roughly divided into hydrothermal vents, cold seeps, seamounts, hadal trenches, mid-ocean ridges, and other habitats (Bian et al., 2010; Orcutt et al., 2011). Microorganisms are at the base of the food web for deep-ocean organisms and drive abyssal biogeochemical cycles (Choy et al., 2017; Fenibo et al., 2023). Microbes, such as bacteria which are susceptible to phage infection, have been increasingly recognized as major ecosystem players because they are abundant and infect organisms that form the basis of ocean biogeochemical cycling (Suttle, 2007; Steward et al., 2013; Moniruzzaman et al., 2017). However, a substantial body of work in marine phagology has often evaluated phages from the epipelagic and mesopelagic zones, while the bathypelagic zone has received less attention (López-Pérez et al., 2017; Jian et al., 2021). Little is known about phage diversity and the mechanisms by which they interact with their host communities in the deep ocean. A recent study revealed that phages from the bathypelagic zone have a unique genetic repertoire, demonstrating the limited nature of our understanding of phages in the largest marine ecosystem, the deep ocean (Coutinho et al., 2023). Furthermore, bathypelagic ecosystems have higher virus-to-prokaryote ratios (De Corte et al., 2012; Lara et al., 2017). In addition, the taxonomic and functional composition, cell densities, and activity levels of the microbial community in the bathypelagic zone are different from shallow and other ecosystems and even vary among different bathypelagic ecosystems (Acinas et al., 2021; He et al., 2023). These differences affected the community and functions of phages. It is necessary to explore phagology in the bathypelagic zone to complete our knowledge of phage diversity and the interaction between them and their host.

In this study, bacterial 16S rRNA sequencing and viral metagenomic sequencing data collected from different bathypelagic ecosystems, including ocean basins, hydrothermal vents, mid-ocean ridges, cold seeps, and hadal trenches, were analyzed to investigate the interaction between phages and their host. The results indicated that the structure of bacterial and bacteriophage communities varies from samples at different locations, even within the same bathypelagic ecological type; however, the dominant species composition in the same type of samples was relatively consistent. The coexistence patterns of bacteriophages and bacteria in cold seeps and hydrothermal samples were distinct compared with other abyssal ecosystems; however, the specific mechanisms need to be further studied. To sum up, this study provided new insights into the characterization of co-occurrence patterns and the interaction between phages and bacteria.

## Materials and methods

### Data analysis of bacterial 16S rRNA

The paired-end reads were overlapped to assemble the V4-V5 tag sequences of bacteria using the Flash program. After the removal of primers, spacers, low-quality fragments, and sequences shorter than 50 bp, the remaining sequences were denoised and screened for chimeric sequences with the pre.cluster command and chimera.uchime command in Mothur software (Schloss et al., 2009). The candidate sequences were classified into operational taxonomic units (OTUs) based on a 97% sequence similarity using the Usearch program (Quast et al., 2013). A representative sequence for each OTU was annotated with threshold 0.8 using UCLUST v1.2.22q by searching the SILVA database. For comparisons between samples, the OTU abundances were normalized by the number obtained from the sample with the lowest counts.

### Pre-processing of viral metagenomic data

The raw reads of viral metagenomic sequencing obtained from the database were trimmed after removing the 5′ end containing non-A, G, C, and T bases. During the process of trimming, sequences of adapters, end of reads with low sequencing quality (value <20), reads that contained a proportion of N reaching 10% were trimmed successively. Then, the sequences < 75 bp in length were discarded and clean reads were obtained. MetaSPAdes 3.12.0 was used to assemble the clean reads followed by gene prediction through METAProdigal (http://code.google.com/p/prodigal/) (Hyatt et al., 2012; Nurk et al., 2017).

### Identification of viral contigs and viral operational taxonomic units

Viral contigs with a length more than 1,500 bp were recovered from metagenome assemblies using VirSorter v1.0.5 and VirFinder v1.1, based on the following criteria: both identified by VirSorter categories 1–6 and VirFinder score ≥ 0.7 and *P* < 0.05. The viral contigs identified by VirSorter and VirFinder were further validated using VIBRANT (v1.2.1, virome mode) (Roux et al., 2015; Ren et al., 2017; Kieft et al., 2020). The identified viral contigs from each assembly were then compiled and clustered at 95% nucleotide identity using MUMmer 4.0 to produce viral OTUs (vOTUs) (Marçais et al., 2018).

### Viral taxonomic annotation

In order to annotate taxonomy of vOTUs, the open reading frames (ORFs) were predicted by Prodigal, as previously described (He et al., 2023). Then, the amino acid sequences of ORFs were used to identify taxonomy of vOTUs using BLASTp (BLAST Version 2.2.28+, http://blast.ncbi.nlm.nih.gov/Blast.cgi) (*E*-value of < 0.0001, bit score ≥ 50) (Castelán-Sánchez et al., 2019; Gregory et al., 2019). Species annotation was obtained from the taxonomic information database corresponding to the NR database. Subsequently, the abundance of each species was calculated using the sum of the corresponding gene abundance of the species. The abundance of species in each sample was counted at the taxonomic levels of domain, kingdom, phylum, class, order, family, genus, and species.

### Host prediction

The hosts were collected from the host annotation of the viral RefSeq database. Additionally, oligonucleotide frequency (ONF) method was used. In the processing, VirHostMatcher v1.0 was run with default parameters, with d_2_^*^ values ≤ 0.2 being considered as a match (Ahlgren et al., 2017; Li et al., 2021). To identify a single predicted host for each viral population, hosts predicted by the highest ranking criterion were chosen.

### Correlation analysis

To explore the interaction patterns and co-occurrence patterns of bacteria and phages in the deep sea, the correlation analysis of the predicted hosts and phages was performed. The calculation and analysis of correlation coefficients were performed via R version 4.0.3 (2020-10-10). The calculation method used was Spearman's. The image was generated using ggplot2 (3.3.5) and igraph (1.2.6) (Sun and Zhang, 2022).

## Results

**Figure 1:**
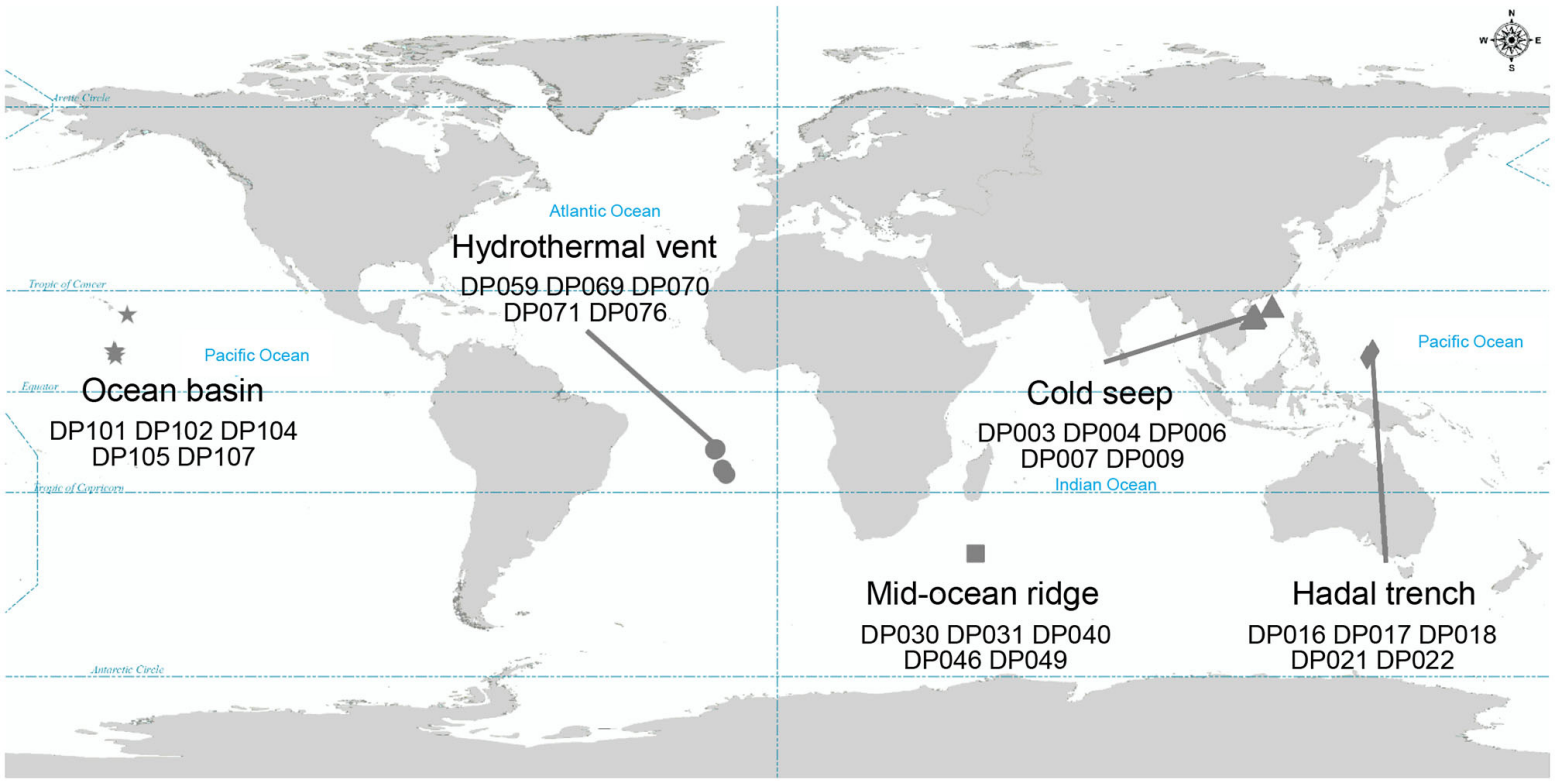
Geographic distribution of samples where metagenomic data were collected. Different symbols represent different bathypelagic environment. Star, circle, square, triangle, and diamond represent the ocean basin (*n* = 5), hydrothermal vent (*n* = 5), mid-ocean ridge (*n* = 5), cold seep (*n* = 5), and hadal trench (*n* = 5), respectively.

To investigate the co-occurrence patterns of microbes and phages, along with the ecological functions of phages in the deep ocean, 16S rRNA gene sequencing and viral metagenomic sequencing public data of 25 deep-sea sediment samples were analyzed (NCBI BioProject ID: PRJNA725024; The National Omics Data Encyclopedia database accession number: OEP002479) (He et al., 2023; Sun et al., 2023). The sampling sites, where sequencing data were collected, included five different deep-sea ecosystems: ocean basin (*n* = 5), hydrothermal vent (*n* = 5), mid-ocean ridge (*n* = 5), cold seep (*n* = 5), and hadal trench (*n* = 5), which are widespread at the Pacific Ocean, the Atlantic Ocean, and the Indian Ocean (Figure 1). The ocean basin is a low zone at the bottom of the ocean, surrounded by relatively high seamounts, and is the main part of the ocean floor (Breyer and Baltar, 2023). Correspondingly, the mid-ocean ridge refers to a series of seamounts with the same origin and similar characteristics that run through the four oceans of the world, and it is the most extensive magmatic system on the Earth (Bennett et al., 2019). The hadal trench is located in the ocean with two steep, narrow walls and a water depth of more than 5,000 m (León-Zayas et al., 2015; Zhao et al., 2022). In contrast to the above, hydrothermal vent and cold seep ecosystems are unique due to their richness in chemical energy. The former, hydrothermal vent ecosystem, is an extreme environment with high temperatures that is rich in many minerals (such as Mn, Fe, Zn, Cu, Pb, etc.) and other chemicals (sulfur, hydrogen, methane, ammonia, etc.) (Dick, 2019). The latter, cold seep ecosystem, is formed by the emission of subsurface fluid into the seabed and is often rich in hydrocarbons (such as methane, oil), hydrogen sulfide, or carbon dioxide (Sun et al., 2021). Anaerobic oxidation of methane is the essential microbial process in the cold seep ecosystem (Beckmann et al., 2021). Despite the extreme environmental conditions, both of these abyssal environments harbor a substantial number of infective viral particles.

### Overview of bacterial communities in the deep ocean

**Figure 2:**
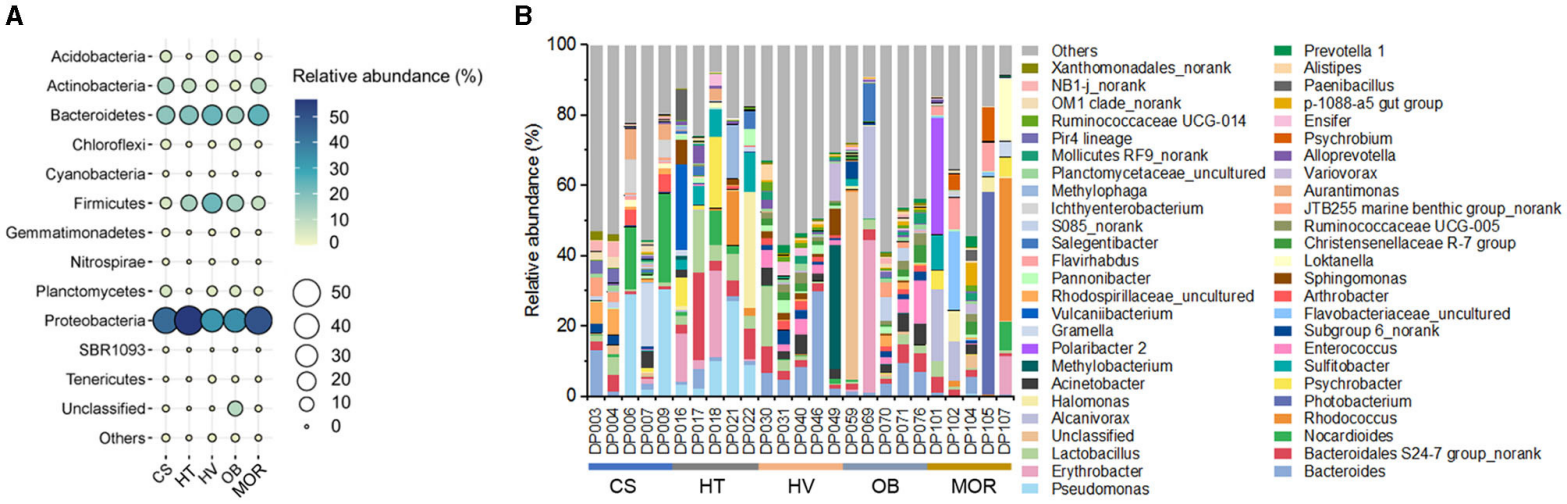
Bacterial diversity of bathypelagic sediment samples. **(A)** Bubble plot of the relative abundance of bacterial phyla according to the 16S rRNA gene sequencing data in five different sediment types. Group names consist of the abbreviation of the bathypelagic environment. The color and size of bubbles represent the relative abundance. The darker the color, and the larger the size, the higher the relative abundance. **(B)** Top 50 bacterial composition of each sediment sample at taxonomic genus level. Different color represents different bacterial genus, and “Others” represent the bacterial genera with abundance < 1%.

To assess the overall bacterial community structure in these sediments, 16S rRNA gene sequencing data of 25 samples were first analyzed for taxonomic profiling. The classification at the phylum level revealed the dominant bacterial lineages to be *Proteobacteria* (on average 43, 56, 32, 34, 50% from cold seep, hadal trench, hydrothermal vent, ocean basin, mid-ocean ridge, respectively), *Bacteroidetes* (17, 19, 23, 15, 24%), and *Firmicutes* (5, 13, 22, 14, 8%) in each environment (Figure 2A). The comparison of bacterial composition in different samples indicated that the diversity of bacterial genera varies in each sample even that were from the same kind of deep-sea ecosystems, displaying a high degree of endemism, which may as the result of each sampling site is far from another and similar to what was found previously in other cold seep microbial communities (Figure 2B) (Ruff et al., 2015). In general, *Pseudomonas, Bacteroides, Erythrobacter*, and *Bacteroidales S24-7 group_norank* occupied a high abundance and were uniformly distributed in each sample (Figure 2B). Notably, deep-sea cold seep and hydrothermal vent ecosystems exhibited more bacterial genera, especially those with an abundance lower than 1%, compared with other deep-sea ecosystems, suggesting that these two environments contained more complex microbial communities (Figure 2B). Both cold seep and hydrothermal vent ecosystems are with elevated microbial activity which were driven by the availability of energy-rich substrates supplied from below, and that may be the reason for the high microbial diversity in these two deep-sea ecosystems.

### Viruses from different deep-sea sediments are diverse

**Figure 3:**
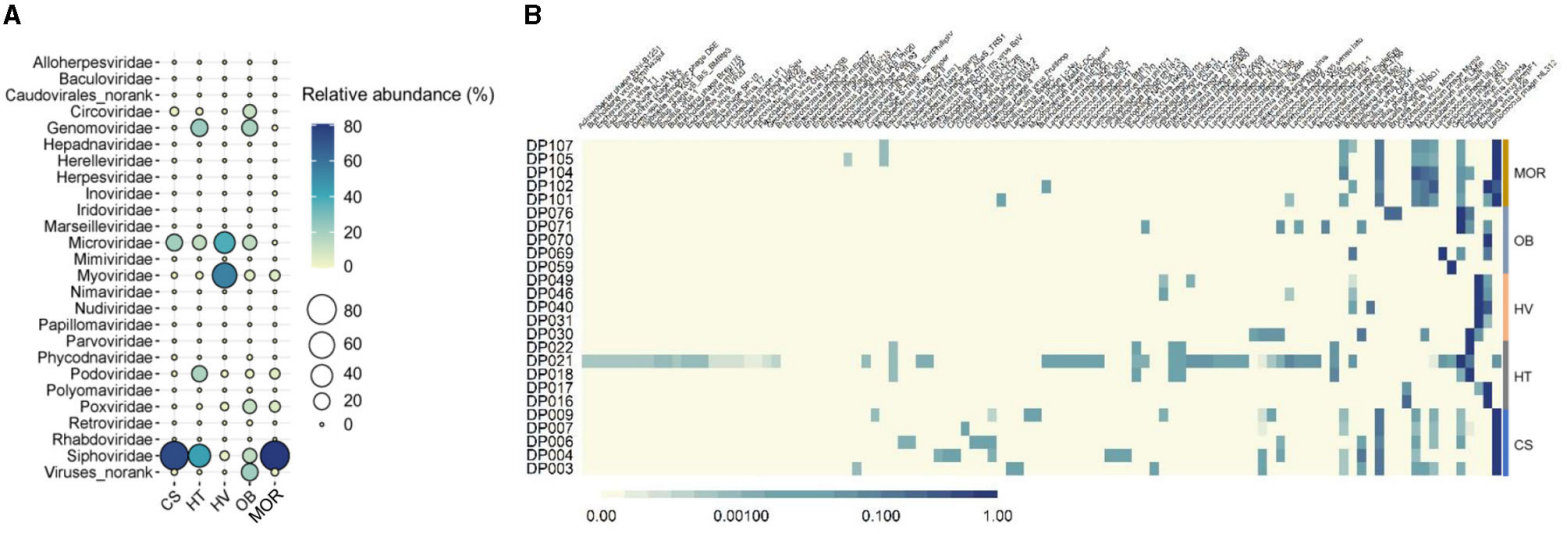
Taxonomic diversity of deep-sea viruses. **(A)** Bubble plot of the relative abundance of viral families according to the viral metagenomics sequencing data in five different sediment types. Group names consist of the abbreviation of the bathypelagic environment. The color and size of bubbles represent the relative abundance. The darker the color, and the larger the size, the higher the relative abundance. “Viruses_norank” represented viruses that were unclassified at the family level. **(B)** Compositions of bacteriophages in each bathypelagic sample. The depth of the color represents the proportion of different phages, and the darker the color represents the more proportion.

From the 25 viral metagenomic data, 420 non-redundant deep-ocean vOTUs were obtained, through manual filtering and clustering. The results of the annotation of the viral family showed that a total of 25 families were classified, while others were classified as norank, indicating that they were viruses that were undetected whether in the laboratory or in high-throughput sequencing before. In all of the viral families, bacteriophages, such as *Siphoviridae, Microviridae*, and *Myoviridae* (47, 13, and 17% in average) constituted the highest proportions (Figure 3A). In addition to bacteriophages, *Genomoviridae* and *Circoviridae*also exhibited high abundance, with the proportion of 8 and 3%, respectively (Figure 3A). Compared to other deep-sea ecosystems, the viral composition of the hydrothermal vent was clearly different. The relative abundance of *Myoviridae* in thermal vents was as high as 54%, whereas the relative abundance of *Myoviridae* was only about 0.6, 1.4, 5, and 5% in cold seep, hadal trench, ocean basin, and mid-ocean ridge ecosystems, respectively, reflecting the uniqueness of deep-sea hydrothermal ecosystems (Figure 3A). As bacteriophages dominated the deep-sea viral community, as predicted above, the composition of phages was further analyzed. The results showed that, at the species level, phages with *Bacillus, Burkholderia*, and *Lactococcus* as hosts had higher abundance (Figure 3B). The composition of the phage community was different among various ecosystems but exhibited relative convergence within the groups (Figure 3B). The diversity of phages was the highest in the deep-sea cold seep ecosystem, consistent with previous findings that cold seeps may be hotspots for viruses (Figure 3B) (Bryson et al., 2015). Furthermore, cold seeps usually have longer geologic history with slower emission of fluids, which provide advantages for the formation of diverse viral communities and complex interactions with hosts (Joye, 2020).

### Phage–host linkages and the predicted host abundance

**Figure 4:**
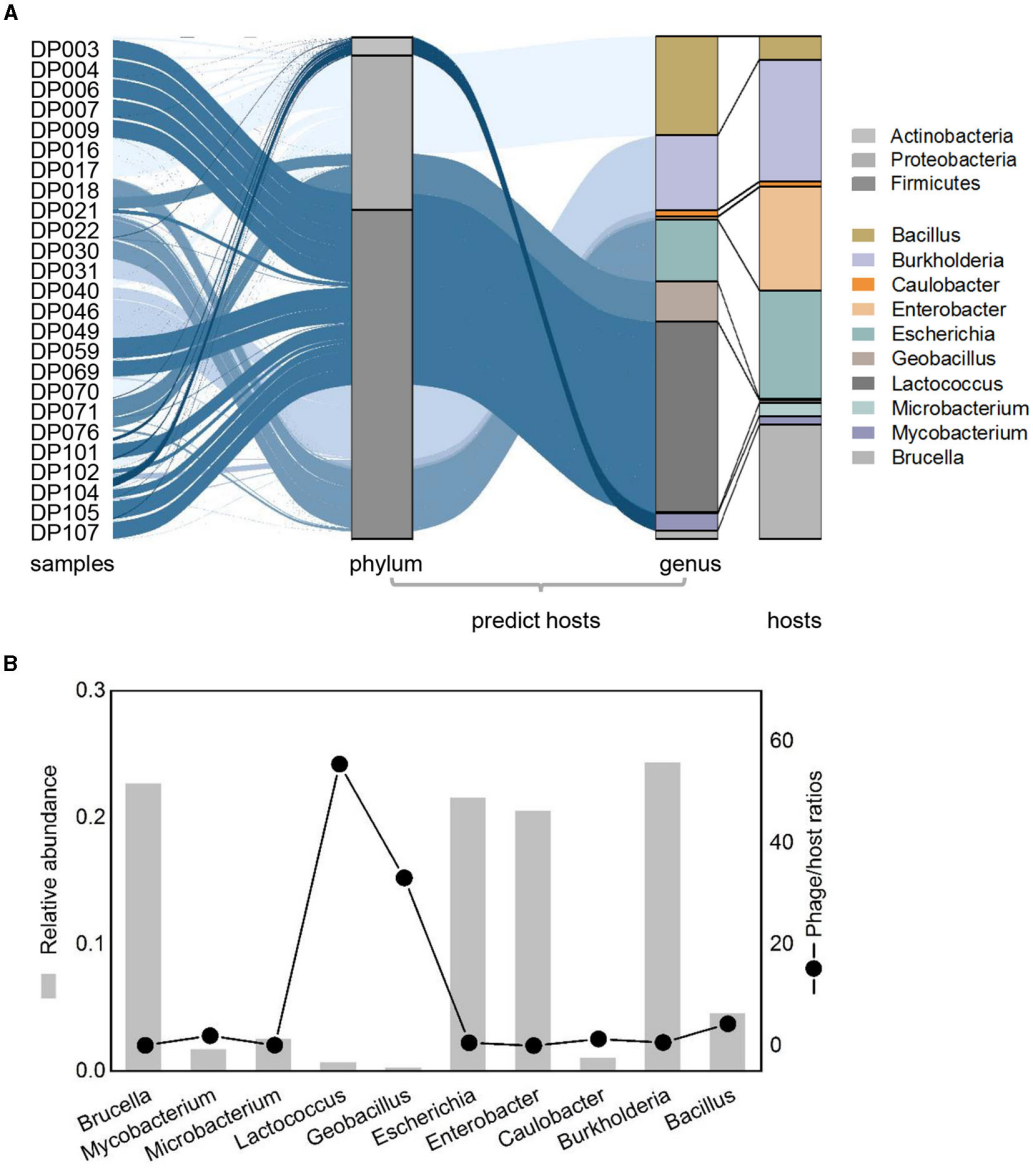
Relative abundance patterns of viruses and their predicted hosts in bathypelagic sediments. **(A)** Comparison of relative abundances of predicted hosts grouped by the host taxonomy at phylum and genus levels. Colored stacked bar chart on the left represents the relative abundance of predicted hosts, whereas the right represents the actual relative abundance of hosts detected in sequencing. Predicted hosts are indicated in the color bar on the right side. **(B)** Lineage-specific phage/host abundance ratios for all predicted bacterial hosts. The left axis represents the actual abundance of the host, and the right axis represents the phage/host ratios.

To investigate the host of detected deep-sea bacteriophage, putative hosts were predicted as previously described (Roux et al., 2015). A total of 90 phages linked to known bacteria hosts, which mainly belonged to three phyla (*Firmicutes, Proteobacteria*, and *Actinobacteria*) and were mainly divided into 10 bacterial genera (Figure 4A). The genera of predicted hosts were dominated by *Lactococcus, Burkholderia*, and *Bacillus* (Figure 4A). Comparing the relative abundance of bacteriophages that linked to predicted hosts with the relative abundance of the corresponding hosts, some differences occurred between them. Some phages had higher abundance compared to their hosts, such as phages of *Lactococcus* and *Bacillus*, suggesting that taxa may be undergoing active viral replication and possibly lysis at the time of sample collection (Figure 4A). The calculation of lineage-specific phage/host abundance ratio for most taxa was from 0.03 to 55, with *Lactococcus* being the highest (Figure 4B). Phages with a high phage/host ratio may be in the period of a high level of active viral genome replication, suggesting that phage lysis may be the main factor of microbial mortality in deep-sea sediments (Figure 4B). Phages with a lower phage/host ratio may have formed prophages in individual hosts and were not freely in the environment, which were not purified from sediments and detected through sequencing, suggesting a complex interaction with host cells in the deep ocean.

### Co-occurrence patterns of bacteriophages and bacteria

**Figure 5:**
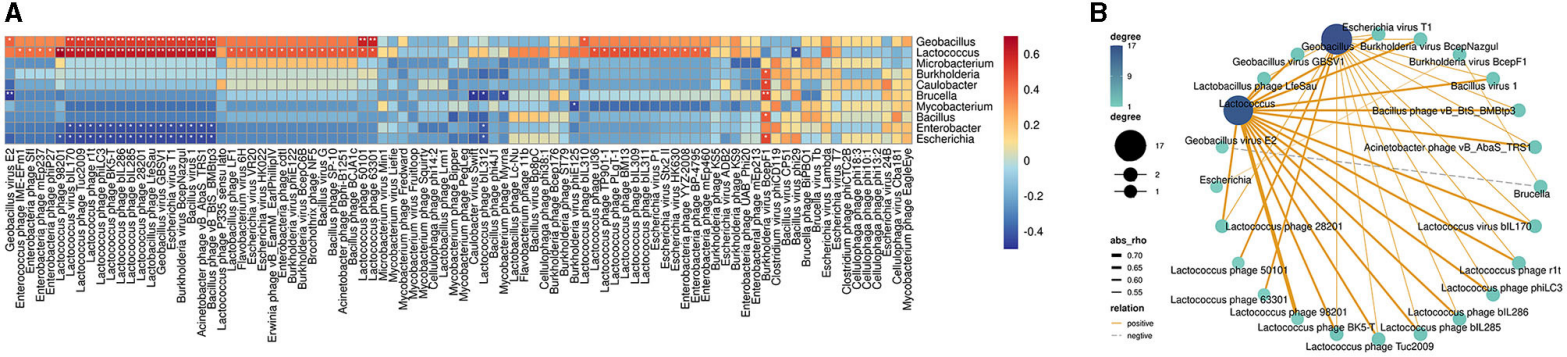
Correlations between bacteriophages and bacteria in bathypelagic sediments. **(A)** Correlation heatmap of bacteriophages and predicted hosts. The predicted bacterial hosts at genus level and all detected bacteriophages in the deep sea were used. The red boxes indicate positive correlations, while the blue boxes show negative correlations. The statistically significant correlations between microbes were indicated with asterisks (*P < 0.05; **P < 0.01). **(B)** Correlation network of bacteriophages and predicted hosts in deep-sea sediment. Line color represents positive and negative correlation. Line thickness represents the strength of the correlation. Dot size/color depth represents the number of related objects.

To further understand how phages correlated with bacteria in bathypelagic sediment, the correlation analysis of the predicted host bacterial genera and bacteriophages was performed. The results showed that the bacterium *Lactococcus* was correlated with more than half of the total phages (Figure 5A). Among them, only one, *Bacillus* virus phi29, had a negative correlation with *Lactococcus*, while others (46 bacteriophages) were tightly positively correlated with it (Figure 5A). Except for *Lactococcus*, another bacterial genus that had strong positive correlation with bacteriophages is *Geobacillus*; it was positively correlated with 19 bacteriophages (Figure 5A). Comparing with correlations of *Lactococcus*, the correlations of *Geobacillus* with phages were weaker (Figure 5B). *Enterobacter* and *Escherichia* showed a relatively strong negative correlation with bacteriophages; they were consistently negatively correlated with 16 phages (Figure 5A). *Brucella* was negatively correlated with four bacteriophages, especially with a tight negative correlation with *Geobacillus* virus E2 (Figures 5A, B). Positive correlations were found between *Burkholderia* virus BcepF1 and five bacteria, including *Burkholderia, Caulobacter, Brucella, Bacillus*, and *Escherichia* (Figures 5A, B). Only the correlations between *Burkholderia* virus BcepF1 and *Brucella* as well as *Escherichia* can be observed in the network, and *Brucella* was in turn negatively correlated with *Geobacillus* virus E2 (Figures 5A, B). In addition, such multiple correlations could also be detected between other bacteria and phages (Figure 5B). Taken together, these findings indicated that the bacteria and phages in the deep sea interacted intricately with each other and played a vital role in modulating the dynamic balance of deep-sea ecosystems.

## Discussion

The bathypelagic zone is characterized by the absence of light, low oxygen, very low concentrations of labile carbon, and higher concentrations of inorganic nutrients (Edwards et al., 2005; Arístegui et al., 2009). Extreme conditions limit the survival of most organisms. The global deep-ocean is dominated by microbial communities that are essential to sustain life in the extreme dark environments (He et al., 2017). Microbial metabolism in the deep ocean is greatly controlled by the phages. They are ubiquitous and can be found in diverse deep-se a ecosystems, such as cold seeps, hydrothermal vents, hadal trenches, and so on (Breitbart, 2011; Sun et al., 2021; He et al., 2023). Currently, the diversity of phages and their interactions with their hosts—such as AMGs encoded by marine phages that are involved in photosynthesis, carbon metabolism, and nitrate reduction, assist the metabolism of hosts, and have a vital role in the biochemical cycle—are widely described in the shallow sea (Thompson et al., 2011; Roux et al., 2016; Breitbart et al., 2018). Little is known about the diversities of phages and the interactions between microbial hosts and phages in the deep sea. In this study, we report extensive examination of the bacterial and phages' diversity in hadal sediment. Our study revealed that different environmental characteristics shape the biodiversity in different deep-sea environments, especially deep-sea cold seep and hydrothermal vent ecosystems, with continuous stable chemical energy as the energy and material resource base of the biosphere, have a more stable and higher biodiversity, both in bacterial and phage composition. The abundance of phages in these two environments was also higher than the other hadal environment. The interaction between bacteria and phages may be more diverse as a result of a stable energy source and the long-term, complex process of biota formation.

As reported, the ratio of phage-to-bacteria is about 10:1; phages are considered to be the main cause of the death of heterotrophic and autotrophic hosts in the ocean owing to their ubiquity and abundance (Suttle, 1994; Breitbart, 2011; Wigington et al., 2016; Breitbart et al., 2018). Phages interact with microbial hosts and other phages in multiple ways, and the interactions with their bacterial hosts and other phages are factors that drive the evolution and diversification of phages and their hosts (Meyer et al., 2012; Betts et al., 2018). In a recent study, it was demonstrated that, over longer timescales, phages and bacteria have evolved more complex resistance and infectivity strategies, along with conserved immunological functions with eukaryotic immune systems (Bernheim et al., 2021; Ofir et al., 2021). However, most findings were obtained from laboratory cultures, and the interaction and coevolution of phages and bacteria in nature were more complex. Since most microorganisms are difficult to culture, it is necessary to seek the interaction from high-throughput sequencing data. Here, through analyzing sequencing data of 25 samples, we revealed the co-occurrence pattern of phages and bacteria in hadal sediment. Furthermore, low phage/host ratios accounted for half of the findings in host predictions, suggesting the prevalence of prophage in deep-sea environments, and indicating a more complex interaction of phages and bacteria in bathypelagic sediment. In addition to this study, a recent surge in viral metagenomic studies (viromics) provides deeper insights into the abundance, taxonomic diversity, and distribution of phages across a wide range of ecosystems (Sunagawa et al., 2020; Peng et al., 2023; Zhang et al., 2023). However, our understanding of phage–bacteria interactions in natural environments is far from being complete, and more studies are needed to fully appreciate how biodiversity and abiotic factors influence phage–bacteria ecological and evolutionary dynamics.

## Data availability statement

The original contributions presented in the study are included in the article/supplementary material, further inquiries can be directed to the corresponding author.

## Author contributions

XS: Data curation, Formal analysis, Funding acquisition, Investigation, Methodology, Resources, Software, Visualization, Writing – original draft. HJ: Supervision, Validation, Writing – review & editing. SZ: Conceptualization, Data curation, Funding acquisition, Methodology, Project administration, Writing – review & editing.
